# Non-Faradaic Electrochemical Detection of Exocytosis from Mast and Chromaffin Cells Using Floating-Gate MOS Transistors

**DOI:** 10.1038/srep18477

**Published:** 2015-12-21

**Authors:** Krishna Jayant, Amit Singhai, Yingqiu Cao, Joshua B. Phelps, Manfred Lindau, David A. Holowka, Barbara A. Baird, Edwin C. Kan

**Affiliations:** 1 Electrical and Computer Engineering, Cornell University, Ithaca, NY 14853, USA; 2 Department of Chemistry, Cornell University, Ithaca, NY 14853, USA; 3 Applied and Engineering Physics, Cornell University, Ithaca, NY 14853, USA

## Abstract

We present non-faradaic electrochemical recordings of exocytosis from populations of mast and chromaffin cells using chemoreceptive neuron MOS (CνMOS) transistors. In comparison to previous cell-FET-biosensors, the CνMOS features control (CG), sensing (SG) and floating gates (FG), allows the quiescent point to be independently controlled, is CMOS compatible and physically isolates the transistor channel from the electrolyte for stable long-term recordings. We measured exocytosis from RBL-2H3 mast cells sensitized by IgE (bound to high-affinity surface receptors FcεRI) and stimulated using the antigen DNP-BSA. Quasi-static I-V measurements reflected a slow shift in surface potential (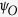) which was dependent on extracellular calcium ([Ca]_o_) and buffer strength, which suggests sensitivity to protons released during exocytosis. Fluorescent imaging of dextran-labeled vesicle release showed evidence of a similar time course, while un-sensitized cells showed no response to stimulation. Transient recordings revealed 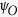 fluctuations with a rapid rise and slow decay. Chromaffin cells stimulated with high KCl showed both slow 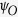 shifts and extracellular action potentials exhibiting biphasic and inverted capacitive waveforms, indicative of varying ion-channel distributions across the cell-transistor junction. Our approach presents a facile method to simultaneously monitor exocytosis and ion channel activity with high temporal sensitivity without the need for redox chemistry.

Synaptic transmission and cell to cell communication in the human body are frequently characterized by the release of charged transmitters and other chemical mediators from secretory vesicles or granules which then impinge on specific receptor molecules expressed on target cells^1^,^2^,^3^. Depending on the excitable nature, the initiating cells respond to chemical inputs by releasing vesicular granules containing specific compounds or by inducing an electrical wave such as an action potential (AP). The process of vesicle fusion with the cell plasma membrane upon stimulation and subsequent release of the granular contents (i.e. in the form of quanta) into the extracellular environment is termed exocytosis^4^. When measured electrochemically such release events reveal a distinctive temporal response^5^,^6^. Exocytosis recordings are also often employed to characterize the mechanism of drug action on cells. For example, amperometric recordings have shown that the Parkinson’s drug L-Dopa increases the quantal size^7^, i.e. the total released charge increases, a consequence of increase vesicle size. There is thus a need to develop high throughput, scalable and multi-functional electronic instrumentation in order to characterize the action of various pharmacological inhibitors, toxins and stimulants on vesicle release. Transmitter and granular release can be specifically stimulated or inhibited depending on the cell type under study. In neurons, electrical excitations in the form of action potentials (AP) propagate along the axon and stimulate neurotransmitter release in the region between the axon terminus of the pre-synaptic neuron and the dendritic spine of the post-synaptic neuron [Fig. 1(a)] called the synapse. The released transmitters impinge on specific receptors on the post-synaptic neuron exciting or inhibiting action potential generation. In immune cells such as mast cells on the contrary, exocytosis can be induced through a receptor effector function where a specific antigen-receptor interaction causes a signal cascade within the cell, culminating in the release of chemical mediators which causes an allergic response. The released compounds from mast cells impinge on cells expressing specific receptors (such as the histamine receptor on smooth muscle cells) [Fig. 1(c)] and elicit a downstream response. In this study we seek to create a CMOS bio-sensor capable of detecting granule release from mast cells as a function of transmitter-receptor induced signaling. We then extend the approach to measuring depolarization induced activity from chromaffin cells where it can function as an electronic post-synaptic sensor [Fig. 1(d)]. Such a system not only provides a test bench for fundamental exocytotic analysis by monitoring release from vesicles and action potential’s with high temporal resolution, which is paramount in understanding cellular kinetics and establishing rapid screening procedures but also sets a promising route towards future artificial synapse systems and ionic-electronic interfacing circuitry.

The rat basophilic leukemia cell (RBL-2H3) is a tumor cell line used frequently as an experimental model for mucosal mast cells^8^. The release of inflammatory mediators from mast cells is the primary event in an allergic response^9^. These cells serve as a robust model for understanding the underlying biophysical and biochemical mechanism which couples signals originating at the membrane receptor with a biological effector function. Immunoglobulins of the IgE class serve as antigenic receptors which are anchored to cells via the membrane protein complex FcεRI. Upon stimulation with multivalent antigen, the receptors crosslink causing a signal cascade within the cell, which eventually culminates in the secretion of preformed mediators stored in the cellular granules. Mast cells form a specialized niche of the immune system, because the triggered cellular activity is immediate. Depending on the particular type of mast cells or basophil’s, secretion occurs within seconds to minutes following the IgE cross linking step. Mast cells also provide a meaningful model for cell activation by an immunological stimulus, i.e., by an antibody-antigen reaction.

We further demonstrate the device detection capability using chromaffin cells of the bovine adrenal medulla to detect neurotransmitter release and related electrical activity. The chromaffin cell is an excitable cell and allows us to study stimulus secretion coupling as mediated by both calcium entry and voltage gated channels, i.e., exocytosis induced by depolarization.

Current methods of monitoring exocytosis include fluorescent techniques^1^,^8^,^10^,^11^, carbon-fiber amperometry^12^ and membrane capacitance measurements^13^. Fluorescence techniques often require labels and sophisticated optics, which increases the complexity of the experiment. On the other hand, amperometry may be prone to noise due to low current levels involved, relies on faradaic chemistry for detection, and is challenging to miniaturize in terms of pixel density, although recent efforts have resulted in significant improvements^14^,^15^,^16^. Non-faradaic transistor-based measurements on the contrary extend the detection capability to electrochemically inactive molecules, are extremely sensitive to surface adsorption, record cellular signals with a high degree of temporal sensitivity, present a naturally occurring high impedance node due to the gate oxide and can render sub-cellular spatial resolution^17^,^18^,^19^,^20^ with very low input referred noise characteristics^21^. Previous work on transistor-based cellular sensing has primarily focused on recording electrical activity from excitable cells such cardiac myocytes^22^,^23^ and nerve cells^21^,^24^. Recently Stern and co-workers^25^ extended transistor based sensing to detect antigen-stimulated T-cell activation detection by CMOS-compatible semiconducting nanowire sensors, however the exact signal generating mechanism was not investigated. In another report, transistor recording of vesicle release from chromaffin cells^26^ was demonstrated using open-gate ISFET’s. The recorded signal was attributed solely to the change in the local pH across the double-layer interface which leads to protonation of the surface and hence a change in surface potential. In this work, we extend this understanding and demonstrate the use of CMOS compatible floating gate transistors as cell based biosensors. Specifically we demonstrate that transistors can detect degranulation from electrically non-excitable cells such as the RBL-2H3 cell line and exocytosis from chromaffin cells. In addition to extracellular pH variation, we provide preliminary evidence suggesting direct molecular binding to the sensor surface as a signal generating mechanism. Since the nature of the FET interface allows one to record both electrochemical and ionic activities simultaneously we demonstrate the detection of AP’s and vesicle release simultaneously. Furthermore, we demonstrate simultaneous quasi-static and impedimetric based signal detection suggesting that FET’s could be used to readout passive membrane properties in addition to secreted charge.

## Cell-transistor coupling

The cell-ISFET (Ion sensitive field effect transistor) interface has been widely investigated in the past^20^,^27^,^28^,^29^,^30^. Typically the cell forms a high impedance seal at the transistor interface and the voltage within the cleft acts as a secondary gate input to the transistor. Changes in ionic activity sets up a potential within the cleft which capacitively influences the transistor output^19^, while chemical changes at the transistor surface such as pH or molecular binding may directly influence the net surface charge^26^. Limitations of this approach include the lack of independent control over the transistor’s operating point. Controlling the ISFET operating point is traditionally achieved by either re-biasing the reference electrode or through source barrier modulation^31^, but applying a large voltage in solution across the cell-transistor interface from a reference electrode may potentially influence or even destroy the cell by electroporation. Also with cells directly immobilized on gate oxide, long-term drift associated with ion penetration into the active region is a serious issue which can potentially lead to current instabilities during measurement. Recent strides in CMOS technology, however allow the use of metal layers and vias to isolate the transistor channel from the sensing region which has shown promising results with very low drift^32^. One drawback, however, is that, top metal interfaces lack chemical specificity to ionic and molecular adsorption, unless specific functionalized coatings are used.

Another class of sensors uses nanowire/nanotube^20^ channels, as opposed to the ISFET’s buried channel, which improves sensitivity due to enhanced electrostatic coupling. The transistor operating point, however, is still modulated by either the reference electrode or a global back gate, which sets a limitation on sensitivity tuning for each individual transistor. This ISFET scheme further imposes restrictions on control circuitry integration. While there have been recent efforts towards creating independent local gate control^33^ to achieve tunability during operation, a highly sensitive, stable, scalable and addressable transducing scheme is still to be demonstrated. Here we demonstrate a fully CMOS compatible, extended floating gate transistor based biosensor for exocytosis. The device permits independent bias control, decouples the sensing region from the active region, and introduces simultaneous charge and impedance readout to record cellular activity.

**Figure 1:**
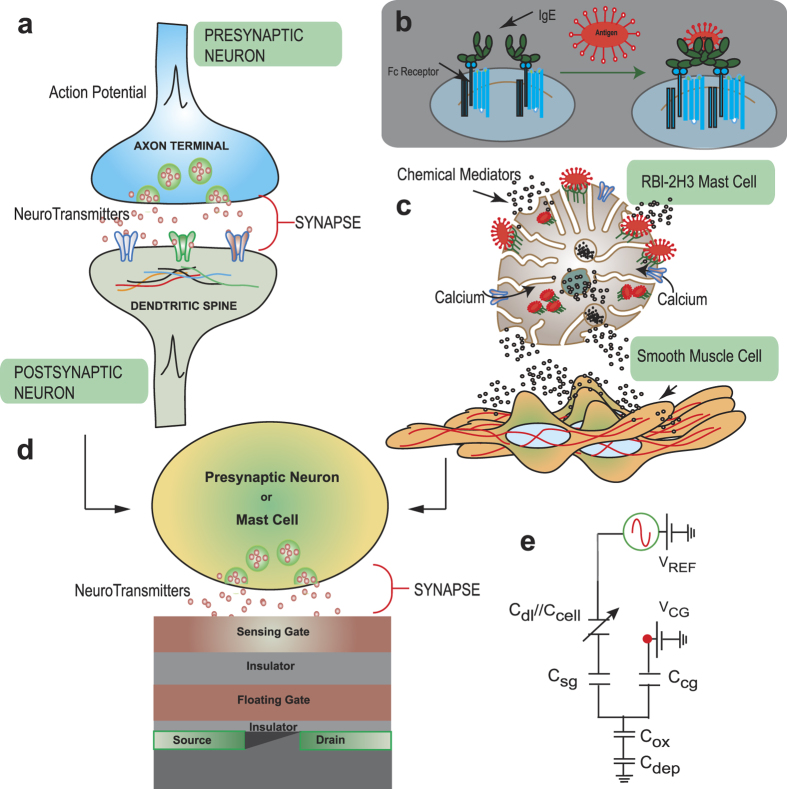
The cell-transistor synapse. (a) Schematic of a neural synapse showing the post-synaptic and pre-synaptic nerve endings. An action potential in the pre-synaptic cell terminates with the fusion of vesicles and release of neurotransmitters (exocytosis) which impinge on the post-synaptic cell receptors. When the intracellular potential of the postsynaptic cell crosses a certain threshold the neuron fires inducing further electrical activity; (**b**) Cross-linking of the IgE upon antigenic stimulation, receptor clustering accelerates degranulation (**c**) Schematic of IgE sensitized mast cell degranulation by antigen DNP-BSA resulting in clear morphological change and release of chemical mediators, which subsequently stimulate smooth muscle cells through a receptor effector function (**d**) Replacing the post-synaptic neuron and smooth muscle cell with the CνMOS effectively creates a cell-transistor biosensor in which the SG effectively serves as an electronic analogue of a synapse and receptor respectively (**e**) Circuit schematic of the CνMOS transistor with capacitively coupled control (CG) and sensing gates (SG) to a common floating gate (FG). The CG and SG serves as threshold weights and after a certain threshold (V_TH_) is reached the transistor turns on.

We previously introduced a MOS sensor termed the chemoreceptive neuron MOS transistor (CνMOS)^34^,^35^,^36^,^37^ for bio-sensing applications (see supporting information Fig. S1). Inspired by the conventional neuron MOS structure^38^, CνMOS has two input gates: control gate (CG) and sensing gate (SG), coupled to a common floating gate (FG) through a 35 nm inter-poly oxide. FG is in turn capacitively coupled to the channel by a 10 nm tunnel oxide [Fig. 1e]. The operating principle is based on weighted sum and threshold operations and the device is widely used in neuromorphic computation^39^,^40^. The capacitive weighting of CG and SG determines the FG potential for the firing signal, i.e., the output drain current. The threshold voltage variability calibrated from the CG is thus a measure of the cell/SG interface condition. In comparison to conventional ISFET’s, each CνMOS can be independently tuned to a desired region of operation and sensitivity using CG. This improves the reliability of recordings, offers new circuit optimization strategies and presents a more flexible biasing scheme in comparison to applying a significant voltage across cells using the reference electrode. The asymmetric capacitive coupling of the CG and SG to the FG further leads to an inherent amplification scheme with high sensitivity to charge and capacitance variations at the SG interface, which can be simultaneously recorded. Conventional floating gate FET sensors do suffer from additional parasitic capacitances, mainly from the extended gate and stray coupling. We designed the CνMOS such that the tight coupling between the transistor channel to the FG, SG and CG ensures an overall bandwidth between 10 kHz and 300 kHz (see supporting information Fig. S3) and the additional parasitic capacitance from the floating gate to substrate does not affect the overall response of the FET to extracellular activity. In addition to the transient current output under a constant bias condition with high temporal resolution, we also demonstrate the unique feature of monitoring the sub-threshold region as an extremely sensitive parameter. Furthermore, the FG can be programmed with either holes (positive) or electrons (negative), which can further interact with the fluid to achieve ionic actuation and detection^35^,^36^, setting the stage for future all-CMOS artificial synapses. Charge injection can also be used for auto-zeroing and translinear operations^41^,^42^, which reduces the burden and complexity of the supporting signal conditioning circuitry required for large-scale integration. To demonstrate the efficacy of the CνMOS transistor as a non-invasive biosensor for exocytosis, we chose to monitor the stimulus elicited response from non-excitable RBL mast cells and excitable chromaffin cells.

## Materials and Methods

### Cell culture and buffer conditions

RBL-2H3 cells were maintained in a monolayer culture in minimum essential medium (Invitrogen Corp., Carlsbad CA), supplemented with 10% fetal bovine serum (Atlanta Biologicals, Norcross, GA), 1 ml/liter mito+ serum extender (Collaborative Biomedical Products, Bedford, MA), and 10 μg/ml gentamicin. Typically, cells were harvested for use 3–5 days after passage. For sensitization, cells were treated with IgE (2 μg/ml) for 1  hour at 37 °C and then re-suspended in buffered salt solutions (BSS: 135 mM NaCl, 5.0 mM KCl, 1.8 mM CaCl_2_, 1.0 mM MgCl_2_, 5.6 mM glucose and 20 mM HEPES, pH 7.4) before dispensing them over native SG surfaces. Experiments were performed at 37 °C using variable pH buffer (HEPES) concentrations ranging between 5 mM and 40 mM in BSS. The cells were stimulated by multivalent antigen DNP-BSA (bovine serum albumin multiply conjugated with 2,4 dinitrophenyl) (0.1 μg/ml). The temperature was maintained through a carefully calibrated air blower. 200 μM monovalent DNP hapten, DNP-aminocaproyl-L-tyrosine (DCT) was used to inhibit the degranulation, serving as a negative control.

Bovine adrenal glands were obtained from a local slaughterhouse and prepared as described elsewhere^43^. Prior to cell immobilization the chips were coated with 0.02% poly-L-lysine (Sigma) and cells were used only within the first two days of cell preparation. Cells were suspended in ringer’s solution containing 150 mM NaCl, 10 mM Hepes, 5 mMCaCl_2_, 5 mM KCl, and 2 mM MgCl_2_ (pH 7.25) (318 mOsm) and subsequently dispensed onto transistors. Ringer’s solution (318 mOsm) with reduced sodium chloride (10%) and n-methyl-glucamine (NMG) (90%) were used to inhibit the occurrence of AP’s. Cells were stimulated by adding high KCl (200 mM) globally to the bath such that final KCl concentration was about 80~100 mM. Experiments on the chromaffin cells were performed at room temperature.

### Fluorescent imaging of vesicle release

RBL-2H3 mast cells were loaded with 2 mg/ml FITC-dextran overnight, 200 μM serotonin and sensitized with 1 μg/ml IgE. On the following day, the cells were washed repeatedly with BSS containing 10 mg/ml BSA before antigenic stimulation^11^. A confocal microscope (Zeiss, Germany) with an immersion lens (60X, oil lens) was used for imaging. The temperature was maintained at 37 °C throughout the experiment using a combination of an objective heater and heated chamber.

### CνMOS detection principles and measurement setup

Please refer supporting information for details regarding transistor geometry [Fig. S1], operation, working principles, instrumentation for quasi-static and simultaneous quasi-static-AC impedimetric operation.

## Results and Discussion

### Quasi-static measurements

**Figure 2:**
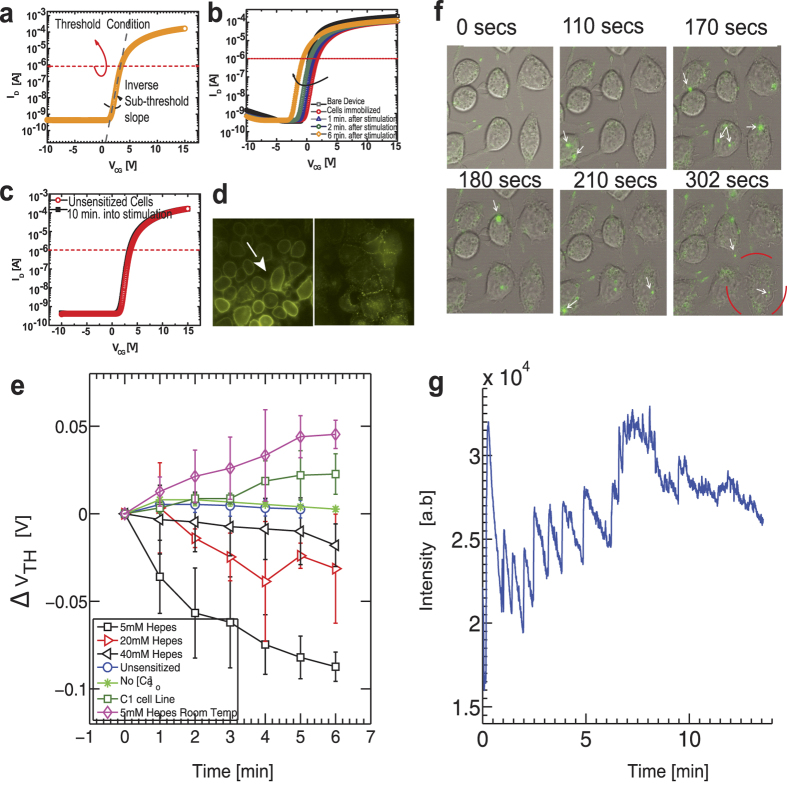
The quasi-static response. (a) Quasi-static IV response of the CνMOS operated from the CG. The V_TH_ is calibrated at constant current of 1 μA, while the subthreshold slope is indicative of capacitance loading at the SG. (**b**) IV response to IgE sensitized mast cell degranulation upon antigenic addition. Notice a clear reduction in V_TH_ as degranulation proceeds with a more positive surface potential evolution. (**c**) Unsensitized cells show no shift in V_TH_ upon stimulation. (**d**) Fluorescent images of IgE-sensitized mast cells (arrow) before (left) and after (right) stimulation. Clustering of IgE receptors is clearly observed along with morphological change. (**e**) Surface potential shifts as function of time after mast cell stimulation with DNP BSA under various conditions. Notice the effects of buffer, temperature and [Ca]_o_. (**f**) Time lapse confocal imaging of FITC-dextran labeled mast cells after stimulation with DNP-BSA. FITC-dextran uptake occurs overnight. Fluorescence is quenched due to the low pH inside the vesicle. Upon release into the extracellular space the fluorescence recovers (green flash). The time stamps reveal a heightened detection of release events (white arrows) a few minutes after antigen addition. (**g**) Energy density indicative of fluorescent intensity for each subsequent time stamp indicates similar kinetics to (**e**).

Antigen-mediated cross-linking of IgE-FcεRI triggers degranulation in mast cells^44^, which eventually results in vesicle release. Cyclic CG voltage sweeps were performed to ascertain the shift in 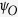 upon antigen addition, as shown in [Fig. 2]. 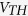 is defined for a constant current level of (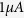) [Fig. 2a]. The readout current and hence 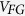 is re-calibrated to this defined level by modulating the CG bias. Any shift in 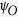 is then reflected by the change in CG bias required to achieve this constant current condition. For example with negative charge released, 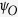 reduces (i.e. surface becomes more negatively charged) which causes 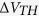 to increase. The IV sweeps (*15* *seconds duration*) indicate larger reductions in 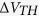 for sensitized cells stimulated with DNP-BSA compared to that of the unsensitized cells [Fig. 2b,c)]. Under quasistatic conditions, the double-layer capacitance is large and 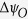 drops mostly across the tunnel oxide as the FG-channel capacitance is the smallest [Fig. 1(e)]. Upon media and cell addition on SG, the subthreshold slope changes immediately in comparison to the bare surface [Fig. 2(b)]. This is due to the additional capacitance *C*_*cell*_ in series with *C*_*dl*_ between the SG and the reference electrode. However, static capacitive loading due to cell immobilization [Fig. 2(d)] or further movement upon stimulation shows no observable effects, reflected in the nearly unchanged sub-threshold slope during the time course of secretion, which not only verifies device reliability but also indicates minute change in *C*_*cell*_ after immobilization and stimulation. Figure 2(e) summarizes 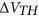 under various experimental conditions. We first stimulated sensitized mast cells under varying extracellular Hepes concentration in BSS, by varying it from 5 to 40 mM. We observed that after stimulation, 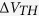 decreased (i.e. 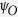
becomes more positive) with time and showed a clear buffer dependence. We explain this dependence as follows. Since the surface of the SG comprises ionizable hydroxyl groups, the interface behaves like a buffer. Hydrogen ions diffuse towards the SG and bind to the exposed hydroxyl charge. The strength of the surface to accept hydrogen ions gives rise to the concept of “buffering capacity” and is strongly dependent on surface equilibrium constants, background ionic strength and surface site density^35^. Introducing additional buffering agent to the solution, in this case HEPES, creates a competition with the surface buffering capacity to negate the surface sensitivity to hydrogen ions. From Fig. 2(e) this would suggest that the acidic environment of the vesicle contributes to variations in 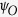. However, the reported signal cannot be totally attributed to pH fluctuations alone. This is because a positive 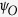 shift of approximately 50–60 mV would imply a change of almost one pH unit in the electrolyte background^35^. Thus non-specific binding of preformed mediators and small molecules that are positively charged are believed to additionally contribute to the 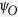 shift. The signal generation mechanism in this case would be similar to that when biomolecules bind to a transistor surface causing a shift in 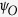 of the order of 10’s of milli-volts^36^,^37^. This result opens up the possibility for highly specific recognition and sensing of released biomolecules (Ex: histamine, serotonin and β-hexosaminidase in RBL cells, tumour necrosis factor-α and Leukotri-enes in mast cells and catecholamine’s in chromaffin cells)^45^,^46^,^47^. Specifically, with surface treatment and immobilization of appropriate molecular recognition elements released molecules can be captured on the sensing surface leading to a signal generation^48^ (similar to an antibody antigen reaction). Nevertheless, since 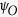 became more positive with time after stimulation and was found to be dependent on the buffer concentration, the physical principle underlying signal generation suggests protonation of the SG interface as one important mechanism contributing to the response.

The absence of [Ca]_o_ was found to suppress any shift in 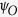 even when cells were sensitized, which strongly suggested that calcium entry was an important precondition to elicit a stimulation response. It is also well known that intracellular calcium oscillations are a pre-requisite for exocytosis to occur^11^,^49^ which will be diminished in the absence of [Ca]_o._ Furthermore, unsensitized cells showed no response upon stimulation, while B4A6C1 mutant cells which are known to weakly degranulate^50^ did not yield any appreciable change in 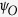 either. These observations together strongly asserted that the observed FET signals were indeed a consequence of granule exocytosis. An important condition imposed during the above study was the temperature of the experimental set up was regulated to be close to 37 °C. When we stimulated IgE-sensitized cells at room temperature under low buffering conditions, the overall 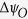 increased slightly with time rather than decrease [Fig. 2(e)], indicative of lower amounts of released “positive charge” and weak degranulation. While this result is promising as physiologically relevant temperatures are important for mast cell exocytosis, the exact signal generating mechanism has not yet been established and requires further investigation. We hypothesize that slow extracellular calcium uptake in the cell transistor cleft is a possible contributing factor. We reason that depletion of cationic charges decreases *C*_*dl*_ in the cell-transistor cleft which leads to reduced screening of SG surface (i.e., “less screened” intrinsic hydroxyl charges on SG), which causes 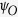 to become more negative.

Confocal microscopy studies of FITC-dextran-loaded mast cells stimulated under identical conditions was performed to confirm the kinetic time scales of the transistor recordings. FITC-dextran, once taken up by the cell, is stored in its secretory vesicles. Due to the low pH within these vesicles, the FITC fluorescence is quenched. The fluorescence intensity increases when exocytosis occurs as the pH of the secreted vesicular content re-equilibrates with the surrounding^11^. Figure 2(f) (white arrows) shows snapshots of vesicular release with green fluorescence transients as a function of time. Figure 2(g) shows the plot of fluorescent intensity as a function of time. Granular release (green), indicated by rapid fluctuations in fluorescence intensity [Fig. 2(g)], is shown to progressively increase with time with a plateau observed after ~4–6 minutes, which is in line with the degranulation kinetics measured by the transistor [black squares in Fig. 2(e)].

### Transient responses at high temporal resolution

**Figure 3:**
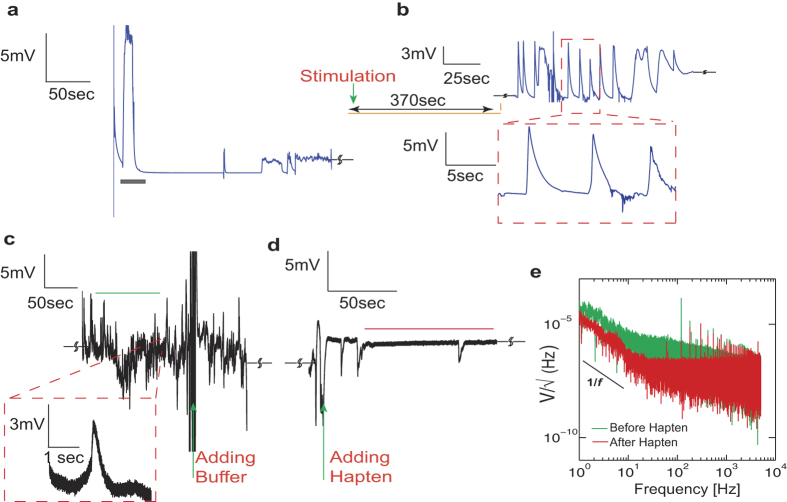
Mast cell transient responses. (a) Immobilized mast cells response to antigenic stimulation by DNP-BSA in BSS. Antigen addition (represented by the grey bar) is followed by a period of inactivity for approximately 2 ~ 3 minutes after which activity begins to ensue. (**b**) Typical transient surface potential fluctuations approximately 5 minutes after stimulation depicts sharp rise and gradual recovery in surface potential over the time course of seconds. (**c**) Adding BSS to stimulated mast cells (green arrow) results in persistent activity. The cells are not displaced during addition of various stimulants. Notice (inset) typical rise and fall patterns in surface potential. (**d**) Monovalent hapten DCT added subsequently (green arrow) to the recording shown in (**c**). A reduction in activity and collapses of the signal to basal noise level is immediately observed. This indicates that a dominant contribution to surface charging is IgE aggregation induced signaling. (**e**) PSD analysis of a 100 second portion of (**c**,**d**) clearly shows a reduction in the overall noise upon hapten addition. A slight reduction in 1/f noise and a more significant decrease in thermal noise indicate that the cell activity which introduces a resistive “cell adhesion” component of noise due to uptake and release of ions and mediators at the interface has reduced.

In the previous section, we described the use of quasi-static surface potential measurements to monitor exocytosis and found that 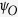 gradually shifts on the time scales of minutes after stimulation. While such recordings prove useful to ascertain antigen/receptor interactions, one cannot monitor events occurring on the order of milli-seconds. Quantal release events with such temporal scales are common during exocytosis^14^, which can potentially be captured with transient recordings under constant CG bias. The release of neurotransmitters and other chemical mediators is accompanied by a low pH cloud and results in rapidly varying electrochemical potentials in the cell-transistor cleft^26^. The proximity of the cell to the sensing surface strengthens the capacitive coupling and increases the cleft resistance, inducing a strong modulation in the drain current during secretion. Due to the pH-sensitive nature of the SG interface, surface protonation contributes significantly to the amplitude of milli-second current fluctuations as corroborated in previous studies^26^. This notion however does not rule out the hypothesis that other chemically active and inactive molecules can bind to the sensing interface and shift 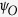 further. During exocytosis, molecules are released within the cell-transistor cleft and diffuse towards the SG surface causing a shift in 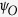. This is similar to a voltammetric signal; however electrochemical redox charge transfer is not involved in this process. Transient recordings of RBL mast cell stimulation revealed a sharp rise and decay within minutes of stimulation (stimulation indicated by grey bar) with DNP-BSA [Fig. 3a]. It is important to point out that mast cell exocytosis ensues only with sustained intracellular calcium oscillations^11^,^49^,^51^. These oscillations take a finite time to initiate and could possibly contribute to the initial delay observed between stimulation (grey bar) and the onset of vesicle release. However, once these oscillations set in, exocytosis should ensue with a distinct temporal behavior. In [Fig. 3(b)] we depict such a trace of activity from a different batch of cells approximately 6 min after stimulation where rhythmic patterns of surface charging are observed. This rhythmic rise and fall in 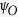 rides on the DC operating point, i.e. it is a transient effect as opposed to the quasi-static threshold voltage measurements described in Fig. 2(e). We do point out that 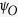 is different between the start and end of the trace, possibly due to pH differences or molecular binding. The rapid re-equilibration in 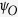 [Fig. 3b] however, is explained as follows. Vesicle fusion is accompanied by protons, ions and a variety of transmitters as previously mentioned. Protons bind directly to the surface hydroxyl groups and shift 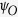 gradually^35^,^37^ establishing a change in surface charge (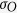). A change in *C*_*dl*_ however due to released ionic charges and compounds causes a transient change in 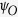 (Eq. 1), which will further shift the surface proton concentration (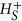) through the Boltzmann relationship (Eq. 2). The surface, which acts like a proton buffer, will then try to maintain 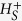
depending on the bulk *pH* condition and re-equilibrate to maintain 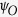 satisfying the condition 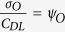
52. If the surface buffering capacity is weak, the re-equilibration will be slow leading to a delay in equilibrium establishment. Hence ionic charges and pH independently contribute to the observed signals.


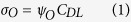



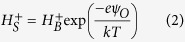


Absence of [Ca]_o_ in BSS suppressed these current fluctuations (not shown) while the introduction of monovalent hapten, DCT, which is known to disaggregate IgE-FcεRI clustering caused by multivalent DNP-BSA, effectively reduced the fluctuation to the baseline. This strongly indicates that the recordings are correlated with IgE cross linking induced signalling^8^. Figure 3c shows the effect of adding BSS to a section of an antigen stimulated response (shown by green arrow). The activity persists without any reduction in amplitude. We then add monovalent DCT indicated by the green arrow [Fig. 3d] and immediately find that the overall signal reduces in noise and amplitude. With uncorrelated noise sources, the total noise density in the system is the sum of individual noise densities. Power spectral density (PSD) analysis [Fig. 3e] performed on 100 second chunks of recordings (green and red bars in [Fig. 3(c,d)]) clearly indicates a reduction in the energy density after DCT addition. Since the increase in noise is decided by the cell-transistor cleft activity, i.e. resistance and diffusion of ions^21^, the noise reduction upon hapten addition directly relates to lower receptor aggregation and ion flow. The *1/f* noise reduces only slightly and appears to be dominated by the transistor channel noise due to small reduction in the transconductance values.

### Signal amplitudes and surface charging

Taking account of both the quasi-static and the transient responses, we attempt to determine the physical basis of the detected signals. Using the simple relationship, 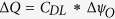 the net total charge difference at the interface can be estimated. With an extracted capacitive ratio of ~12 and a specific double layer capacitance of 16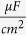, 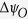 of ~5 mV corresponds to a net change in overall interface charge of 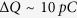. We do point out that the calculation presented is a conservative estimate for surface charging and does not include direct molecular binding. Vesicle size in RBL-2H3 cells are approximately 0.4 μm^53^ in diameter. If each granule contained ~0.05 pC and a total of 100 vesicles were released during exocytosis, the total charge secreted (~5 pC) would correspond to mV signal amplitudes. It also suggests that the degranulation response is derived from a collection of vesicles rather than single vesicle events. On an average we have ~10–15 cells covering every SG.

### Impedance spectroscopy at the RBL cell-transistor interface

As secretory granules fuse with the plasma membrane during exocytosis, the overall cell area increases in proportion to the extruded vesicular surface area. It is well known that the most biological membranes have a specific capacitance of ~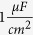 and hence an increase in membrane area directly reflects an increase in overall capacitance^54^. RBL mast cell degranulation is often accompanied by rapid membrane ruffling and morphological changes^55^ within minutes of antigenic stimulation, resulting in a slight increase in total membrane capacitance reaching ~0.5 pF^56^,^57^. This is in stark contrast to traditional rat mast cells which reveal a near ~30pF change^58^. The capacitance is also affected by the number of granules which is lower in the RBL line. This capacitance change associated with granule fusion is normally captured by patch clamp capacitance recordings. Time-resolved impedance measurements thus allow for simultaneous measurements of membrane capacitance and conductance, thus providing a powerful tool to detect such secretion coupling events.

**Figure 4:**
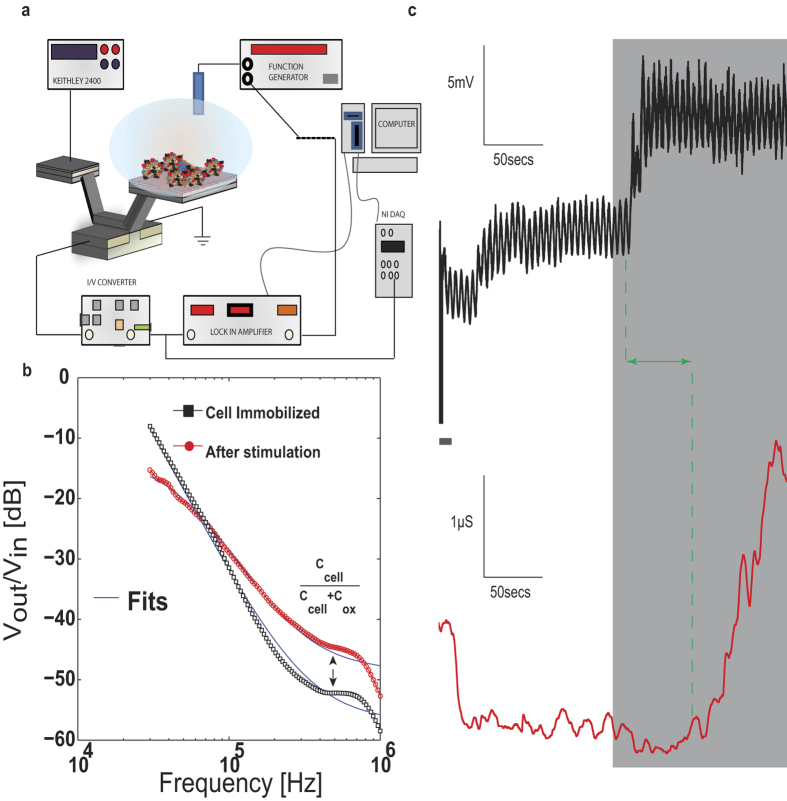
Simultaneous Impedance and Charge detection. (a) Measurement setup showing the simultaneous impedance and charge detection by the split excitation technique on the CνMOS. The CG delivers the DC excitation while the reference electrode delivers the AC small signal (0.1 V). The AC impedance magnitude signifies the transconductance as a function of frequency. (**b**) The pole-zero (Bode) responses before and after stimulation show the zero moving in, which is possibly due to the increase in cell membrane area during exocytosis. By a crude fit, we extract an overall increase in capacitance of ~0.1 pF, which includes the capacitance increase from all the cells immobilized on the surface. The shift in the first pole position is due to an increase in interface resistance and the shift in zero is mainly due to capacitance changes at the cell transistor interface. (**c**) Simultaneous surface potential and transconductance measurements by measuring the DC and AC components (at 40 kHZ) independently. Notice that as soon as stimulation is initiated, there is a slight delay in response after which shifts in surface potential are observed (upper). A concomitant increase in g_m_ and hence capacitance is also observed (bottom) although there exists an initial decrease during stimulation. The change in capacitance shifts the transistor g_m_ by ~1 μS.

In order to further corroborate cell stimulation and exocytosis detection, we sought to confirm the overall capacitance shift through impedance analysis. Impedance detection along with surface potential was performed using a split excitation technique [Fig. 4a] where the AC signal is applied through the reference electrode and the DC bias through the CG (see Supporting Information for details). In order to concomitantly measure charge and net impedance, the TIA output was split two ways with one end fed to the NI DAQ board sampling voltage at 10 KHz while the other end was fed to the LIA. By monitoring the transconductance 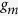 as a function of frequency, one can measure the fluctuations in capacitance and interfacial resistance^59^ as a shift in the pole-zero response [Fig 4b] upon mast cell immobilization. Previous efforts with the ISFET^60^ used the AC impedimetric change to ascertain the seal resistance in the cell-transistor cleft, while in this paper we employed the approach to capture fluctuations in cell/transistor interfacial impedance away from the quasi-static regime. The impedance readout at this operating point mainly depended on the net capacitance, and much less on charge fluctuations at the interface. The model for the cell-SG interface in frequency domain follows a similar theory to biomolecular modeling under frequency analysis^36^ with the first pole dominated by interface resistance and the first zero dominated by the cell passive properties (see supporting information Fig. S2 and Fig. S3). In [Fig. 4b] we show the effect of antigen stimulation on the Bode response and observe a shift in the zero indicating an increase in the cell capacitance after stimulation. This result is consistent with the overall simulations and models presented earlier^36^,^59^,^60^. We fit the data in spite of the limited frequency range to gain an estimate for circuit parameters. We extract a capacitance change between ~0.05 pF–0.1 pF, depending on selection of fitting parameters. This is lower in comparison to the whole-cell patch clamp recordings of mast cell exocytosis 57 where shifts on the order of 0.4 pF have been observed. We attribute the smaller change in impedance to the partial attachment of the membrane in the cell-transistor overlap region, which changes less in surface area than the larger free membrane (portion away from the transistor surface) during exocytosis. Moreover the impedance change is also affected by total cell coverage. We do point out that in a recent impedance study^59^, although performed on a different cell line, the extracted values of seal resistance and cell capacitance agree well with the values reported here.

In [Fig. 4c] we perform a concomitant 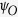 and impedance measurement at a fixed frequency (40 KHz). We find a step increase in 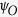 upon antigenic stimulation with a distinct time course indicative of surface charging. The recording is not high pass filtered in this study so as to reflect this DC shift. The AC impedance reflected by the transconductance measurement initially decreases upon stimulation but then subsequently increases (shaded region), suggesting cell secretion and capacitance change due to morphology. We do observe about 50-second delay between the surface charging and impedance change, possibly reflecting differences in dynamics between cell secretion and gradual membrane expansion. Although performed on a population of immobilized cells, this experiment indicates that transistors can be used to simultaneously measure capacitance (impedance) and charge (surface potential). Future experiments will be aimed at experiments involving single cell studies.

### The Chromaffin cell-transistor coupling

To further demonstrate exocytosis detection by CνMOS with high-resolution transients, we chose the chromaffin cell of the bovine adrenal medulla as a another established exocytotic model^61^,^62^, albeit through a different mode of stimulation. The chromaffin cell helps serve as a model system of voltage-gated ion-channel activity and exocytosis induced by membrane depolarization. Chromaffin cells are known to secrete catecholamines as a consequence of exocytosis and the granular content is known to be highly acidic which should contribute to a net shift in surface potential upon release. One important difference between the chromaffin and mast cell studies is that the electrical currents in the form of AP can flow in the cell transistor junction as a result of activated ion channel activity in neuro-endocrine cells^63^,^64^. In previous transistor-based studies of chromaffin cells, this aspect of signaling (i.e. AP’s) was ignored^26^.

**Figure 5:**
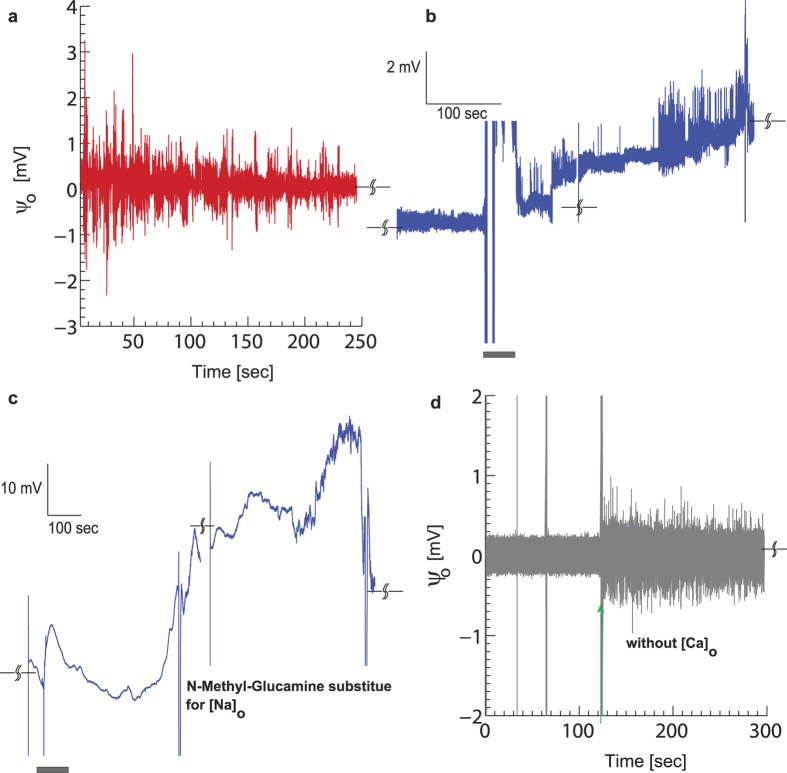
Transient responses of chromaffin cells. (a) Sample of activity after high KCl induced depolarization (high pass filtered) shows rapid fluctuations in surface potential, which suggests AP with peak-peak amplitudes reaching ~2 mV. (**b**) Effect of adding Ringer’s solution rich in [Na]_o_ to the transistors with cells previously bathed in NMG substituted Ringer’s and stimulated with high KCl. Notice the steady shift in surface potential (not high pass filtered) indicates positive secreted charge along with rapid spikes resembling AP, suggesting that the transistor response is closely tied with [Na]_o_. (**c**) A 300-second recording of stimulated activity (not high pass filtered) in the presence of NMG substituted Ringer’s shows clear increase of surface potential shifts with time, but AP’s are reduced. (**d**) Stimulated response of chromaffin cells in the absence of [Ca]_o_. The presence of AP persists.

Chromaffin cells suspended in ringer’s solution were dispensed on the poly-l-lysine coated SG. After about 45 ~ 60 minutes concentrated KCl solution was added with a pipette to reach a final KCl concentration of ~80 mM. Upon KCl stimulation which causes membrane depolarization, we observed sharp fluctuations in readout current [Fig. 5a]. The recorded signals were found to be strongly dependent on extracellular [Na]_o_ and [Ca]_o_, as expected for contributions from APs and catecholamine release. We independently confirm the presence of APs and exocytosis through the following control experiments. Figure 5b depicts the effect of [Na]_o_ replenishment by adding ringers solution rich in [Na]_o_ (marked by grey bar) to cells previously stimulated by high KCl in NMG substituted ringers media. The high-pass filter criterion was removed to observe slow 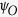 shifts. We immediately observe surface charging and rapid fluctuations upon [Na]_o_ being introduced indicating that fluctuations are a true consequence of AP activity. In another recording [Fig. 5c] we once again add high KCl to immobilized chromaffin cells in NMG rich ringer’s media. We notice a gradual increase in 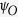 as a function of time upon KCl depolarization whereas APs are not evident. The rise in 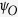 is consistent with a net positive surface charging effect similar to the mast cell degranulation study, indicating that exocytosis is possibly being detected. We do observe fluctuations in 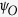 on a slow time scale (~100 msec) typical of delayed surface re-equilibration (not shown). In the absence of [Ca]_o_, 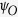 shifts were absent altogether (not shown) while AP waveforms characterized by their milli-second time scales and rapid activity continued to persist [Fig. 5d]. This result suggests that the 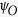 shifts measured previously may be a consequence of exocytosis. Compared to Fig. 5a, the observed amplitude of extracellular APs in the absence of [Ca]_o_ [Fig. 5d] were smaller over ~3 experimental runs, although we present only one representative result here. This reduction could be due to the contribution of voltage-gated Ca currents to the AP but this hypothesis requires further experimental investigation.

**Figure 6:**
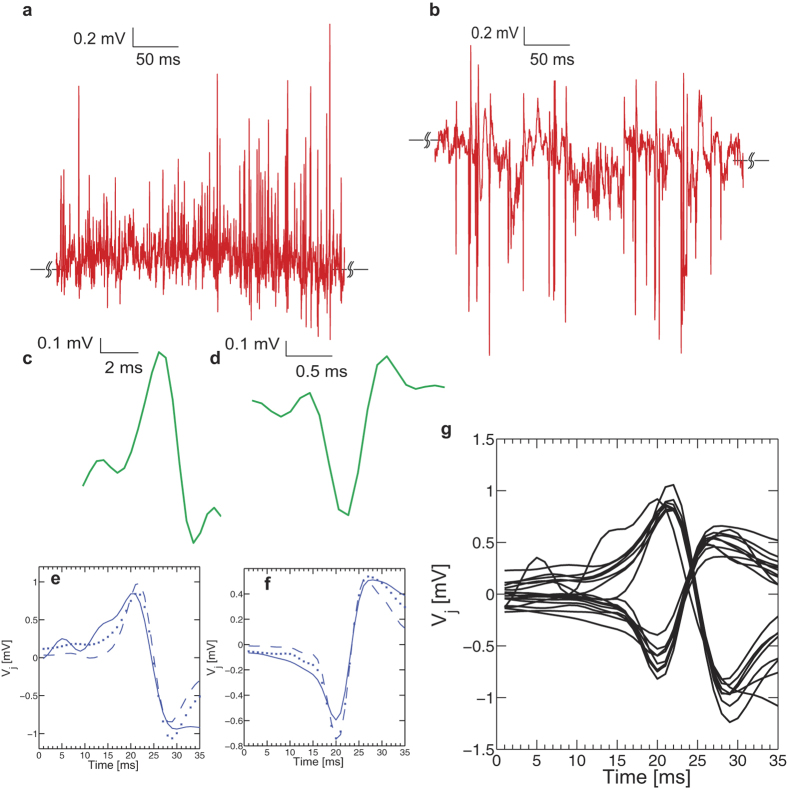
Action potential characteristics. (**a**) Transient activity for chromaffin cell stimulation depicting biphasic waveforms observed during the rising phase of an intracellular AP. (**b**) A trace of inverted capacitive waveforms. (**c**,**d**) Templates of the biphasic and inverted AP waveforms used for matched filtering. (**e**) Average match filter response for 3 independent experiments shows the shape and amplitude of the biphasic response recovered. (**f**) Inverted capacitive response for the same. (**g**) Clusters of biphasic and inverted waveforms after performing an amplitude threshold and match filter operation. The shape and amplitudes of the waveforms are very homogenous and amplitudes lie between 0.8–2 mV peak to peak.

**Figure 7:**
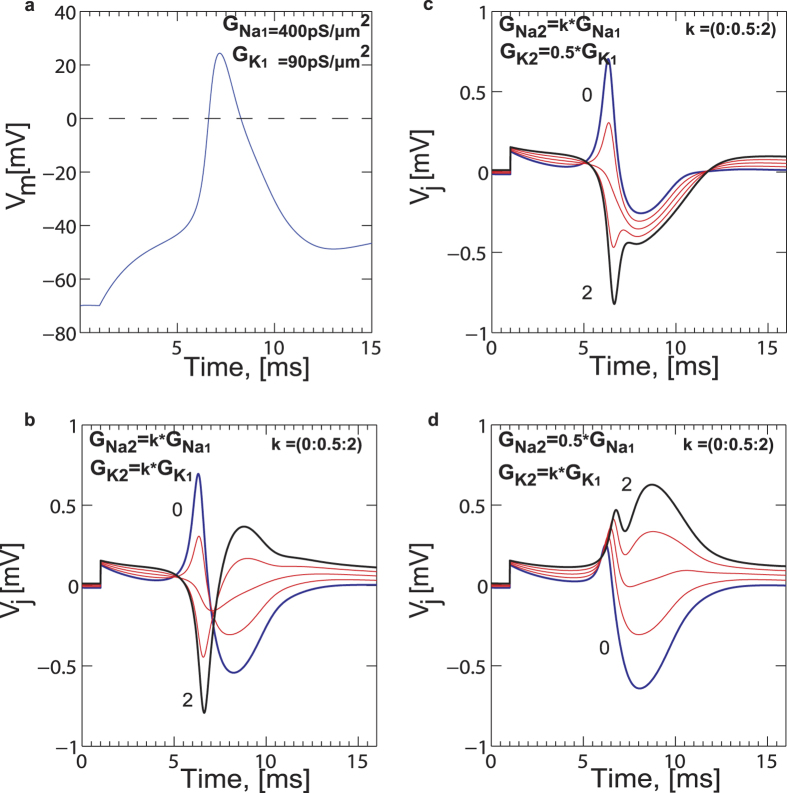
Simulation of electrical response using the point contact model. (**a**) A typical intracellular membrane voltage when an AP is elicited. (**b**) Effect of raising the overall junction conductance with respect to the free membrane conductance. Notice, that when conductance values for both Na^+^ and K^+^ are simultaneously raised in the junction, the extracellular waveforms shift from biphasic to inverted capacitive. (**c**) Similar operation to (**b**) with the K^+^ conductance in the junction decreased with respect to the free membrane. When the Na^+^ and K^+^ conductance in the junction is now raised, the Na^+^ activity becomes much larger than the K^+^ activity. This causes a trough in the AP waveform. (**d**) Similar operation to (**c**) but with the Na^+^ conductance decreased in the junction. This causes an intracellular-like waveform although with a diminished amplitude.

The effect of ion channel distribution between the free and transistor-attached parts of the cell membrane is a key parameter that determines the amplitude and shape of the AP. As shown in [Fig. 6a,b], we observe both biphasic and inverted capacitive responses [Fig. 6c,d]. Such waveforms are classically interpreted^65^ as (a) a capacitive (i.e. biphasic) response across the cell membrane due to the intracellular AP which gives rise to a shift in junction voltage (*V*_*j*_) and (b) the ion-channel conductance in the cell-transistor cleft (inverted capacitive) of either the Na, K or both are raised against the free membrane. We perform match filtering and amplitude threshold signal processing on three independent experiments lasting between 200–300 seconds each where chromaffin cells are stimulated by high KCl. Over the time course of the entire experiment, recurring AP waveforms with a clear biphasic and inverted capacitive response are observed [Fig. 6e,f]. The inverted response has on average slightly lower amplitudes in comparison to the biphasic response. Figure 6(g) depicts a cluster of the biphasic and inverted waveforms for ~100 seconds of recorded data after match filtering and threshold operations. In order to elucidate the physical basis of the waveforms (i.e. shape) we used the point-contact model including the Hodgkin-Huxley (HH) description for ion channel activity in the cell-transistor cleft developed previously^65^ (see supporting information, Fig S3(b)). Following the approach used in^65^, the intracellular membrane potential 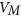 elicited through a current stimulus was calculated using Eq. (3) [Fig. 7a] along with the rate equations for Na^+^, K^+^, Ca^2+^ and Ca^2+^ dependent K^+^ conductances pertinent to chromaffin cells^66^ at room temperature. 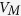 was then used to calculate the cleft potential 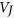
in Eq. (4).


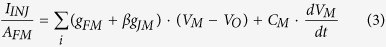



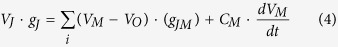



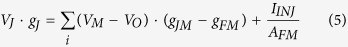


The main assumption made here is that dynamics of the trans-membrane and extracellular voltages are coupled. The cell membrane creates a potential 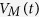 (Eq. 3) and the cleft reacts with a signal 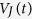. By substituting Eq. (3) in Eq. (4) we achieve Eq. (5) which depicts the interplay between the free and attached membrane conductances. Here 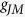 represents the conductance in the cell membrane in contact with the transistor SG, 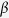 represents the ratio of immobilized membrane surface area to the free membrane area, 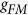 represents the conductance in the free membrane not in contact with the transistor and 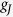 represents the conductance in the cell-transistor cleft due to displaced ions (i.e., the effective seal resistance). 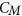 is the specific membrane capacitance and the index *i* indicates the different types of channels (i.e. Na^+^, K^+^ and leakage channels). The cell thus acts like a current source creating an extracellular potential due to the net resistance in the cleft^59^.

In the model we chose to maintain the specific free membrane conductance for Na^+^ (G_Na1_), K^+^ (G_K1_) and Ca^2+^ dependent K^+^ conductance as 400 pS/μm^2^, 90 pS/μm^2^ and 70 pS/μm^2^ respectively. The reversal potential values for Na, K and leak currents were 50 mV, −70 mV and −75 mV respectively. A cell diameter of ~15 um was assumed. All Ca^2+^ dependent activity was simulated based on the theory outlined in previous studies. The overall attached membrane conductance (G_Na2_ and G_K2_) was scaled by factors ranging from 0 to 2 in steps of 0.5 (i.e. G_Na2_ = G_Na1_·k and G_K2_ = G_K1_·k) with respect to the attached membrane values [Fig. 7b–d]. It is important to note that the values of conductances used here are close to the values used previously for rat chromaffin cells. These values are likely to be different for bovine chromaffin cells used in this study. Although it would be of high interest to elucidate the functional role of each channel type in the overall secretory response, the information at present is not sufficient to model all conductances precisely and merely serves as an approximation of the predicted waveforms. For example the authors in^66^ used an integrated version of all Ca^2+^ channel subtypes to describe chromaffin cell AP activity by modifying the properties of L-type channels in thalamocortical relay neurons^67^. The validity of the model was corroborated against experimental data. We assume that bovine and rat adrenal chromaffin cells have similar ion channel expression and hence can be described by similar rate equations and constants. We find that with this assumption, the recorded amplitudes and waveform shapes are remarkably similar to theoretical predictions.

When the overall ionic conductance in the cleft was raised with respect to the attached membrane with G_Na2_ = k*400 pS/μm^2^ and G_K2_ = k*90 pS/μm^2^, the waveforms resemble inverted transients (k = 2) [Fig. 7b]. We also find that if G_K2_ is lowered in the cleft to 45 pS/μm^2^, GNa_2_ and hence the overall Na^+^ conductance is much higher when scaled than K^+^ in the cleft, which causes the inverted response to become broader [Fig. 7c]. This happens because Na^+^ rushes into the cell during the rising phase of the AP, and ionic charges get depleted in the cleft resulting in a steeper trough in the AP waveform. Decreasing the Na^+^ conductance in the cleft in comparison to the attached membrane however creates a stronger rise in the extracellular AP and a similarly diminished intra-cellular waveform [Fig. 7d], with a slightly broader time course, which is indicative of K^+^ channel dominance. Such waveforms however were found to be statistically infrequent (data not shown). With respect to extracellular AP amplitudes, a cleft conductance of approximately ~5000 pS/μm^2^ which is reflective of a moderate seal resistance, results in extracellular peak-peak amplitudes of approximately ~800 μV–1 mV [Fig. 7b], consistent with previous transistor studies on neurons^19^,^20^. Higher cleft resistances, i.e. a lower conductance, will amplify the extracellular potential even further and hence improve the seal between the cell and transistor, which is paramount to ensure high signal-to-noise ratio. A higher specific membrane capacitance of ~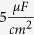 was assumed in the simulation to account for sodium channel gating charge movements^68^ which can transiently enhance the capacitance and increased cell surface area due to multiple cells. A considerable amount of experimental effort has been previously performed on neurons to elucidate the underlying physical basis of such signals^18^,^19^,^69^ based on ion channel re-distribution, enhancement and depletion. From [Figs 6 and 7] we infer that since both biphasic and inverted capacitive responses were recorded by the CνMOS, ion channel re-distribution across the chromaffin cell membrane is an important source of signal and up-regulated Na^+^, Ca^2+^ dependent K^+^ and K^+^ conductance’s in the cleft with respect to the free-membrane as the likeliest scenario for waveform difference. The experimental evidence presented in this study further validates extends the applicability of the point-contact model to primary neuro-endocrine cells.

## Conclusion

Degranulation in mast cells and action potential activity in chromaffin cells were monitored through quasi-static surface potential measurements, high resolution transient recordings and impedance spectroscopy using CνMOS transistors. Surface potential modulation was observed through a) a slow pH dependent shift (5 mV~30 mV baseline shift), b) rhythmic fluctuations on milli second time scales (1–5 mV amplitudes) suggestive of vesicular release events and c) fast extracellular ionic currents indicative of action potentials (~2–3 ms half widths). The FET response upon stimulation was found to agree well with fluorescence data confirming that the transistor was indeed recording exocytosis induced signals. In comparison to existing techniques FET based exocytosis detection does not rely on redox chemistry opening up the possibility of high temporal resolution based detection of transmitters that are electrochemically inactive. Simultaneous impedimetric and quasi-static measurements achieved through a new split gate approach revealed that impedance changes occurred on a slower while surface charging occurred on a much finer time scale. Furthermore, action potentials and secretory responses from chromaffin cells were monitored with extremely high temporal resolution. Action potentials with both a biphasic and inverted capacitive waveform were found to exist and were attributed to different ion channel distributions in the cell-transistor cleft. Future work aims at demonstrating CνMOS arrays coupled to large cell populations with sub-cellular resolution and sensitivity.

## Additional Information

**How to cite this article**: Jayant, K. *et al.* Non-Faradaic Electrochemical Detection of Exocytosis from Mast and Chromaffin Cells Using Floating-Gate MOS Transistors. *Sci. Rep.* **5**, 18477; doi: 10.1038/srep18477 (2015).

## Supplementary Material

Supplementary Information
