# Supplementary material for: Non-Faradaic Electrochemical Detection of Exocytosis from Mast and Chromaffin Cells Using Floating-Gate MOS Transistors
Source: Sci Rep. 2015 Dec 21;5:18477. doi: 10.1038/srep18477 (PMC4685269; doi:10.1038/srep18477)
Supplement: Supplementary Information [file srep18477-s1.doc]

Supporting Information

for

Non Faradaic Electrochemical Detection of Exocytosis from Mast and Chromaffin Cells Using Floating-Gate MOS Transistors

*Krishna Jayant^1*^, Amit Singhai^2^, Yingqiu Cao^1^†, Joshua B.Phelps^1^†,*

*Manfred Lindau^3^, David A. Holowka^2^, Barbara A. Baird^2^, and Edwin C.Kan^1^*

*^1^Electrical and Computer Engineering, Cornell University, Ithaca, NY 14853, USA*

*^2^Department of Chemistry, Cornell University, Ithaca, NY 14853, USA*

*^3^Applied and Engineering Physics, Cornell University, Ithaca, NY 14853, USA*

*† Equal Contribution*

^*^Corresponding Author: [kj75@cornell.edu](mailto:kj75@cornell.edu) ; Tel: +1-908-930-7179

Supporting information outlines the overall operation, set up and transistor transfer function derivation. Additional control experiments for results presented in the main manuscript are also presented.

| 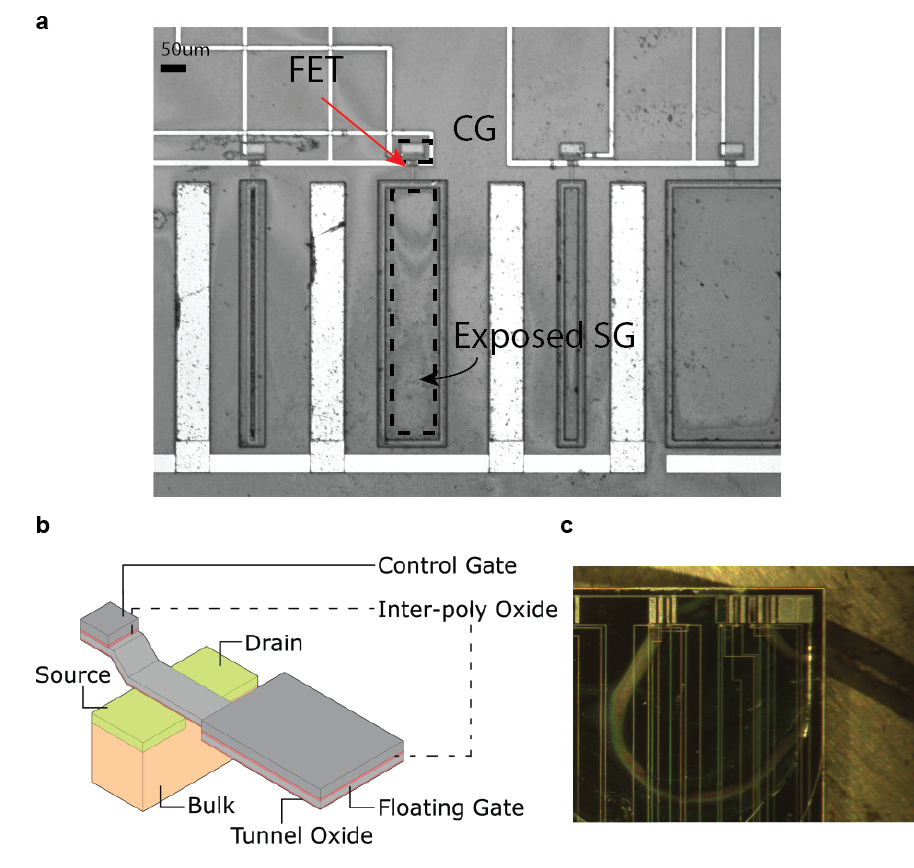 |
| --- |

*Figure S1. (a)SEM of the fabricated chip showing an array of CνMOS transistors with extended FG's. Notice how the transistor is tightly coupled to the extended FG, SG and CG. The design allows for independent tuning of the transistor and the sensing region respectively. (b) Orthogonal cartoon of the CνMOS showing the different poly-silicon layers. (c) photograph of the chip after use. The long leads are connections to the bond pads.*

***Transistors , instrumentation and measurement setup***

The chips were fabricated in a 1.5μm foundry CMOS (AMS) double-poly process with SG exposed and the rest of the chip covered with a 2µm polymer insulation. The first polysilicon layer forms the FG while the second polysilicon layer forms the CG and SG. The SG interface is exposed to bio-media while the CG is buried below insulation. The control oxide thickness (i.e. interpoly oxide) is 35nm, the tunnel oxide (i.e. between the FG and channel) is 10nm, and the capacitance ratio between SG and CG () ranges between 2 and 25 for various designs. SG areas on the chip ranges between to while the CG areas are. The devices have an active channel area of. An epoxy well was realized above the chip to isolate the cells and media from the bond pads.

Three distinct measurement procedures were performed, quasi-static I/V sweeps for monitoring gradual changes in surface potential (), high-resolution transient recordings of extracellular secretory activity, and impedimetric detection of the SG interface. For I/V measurements, we used a semiconductor parameter analyzer (HP 4145 B) with the drain voltage () kept constant at 1V across all measurements. The transient measurements at a fixed CG bias was recorded by interfacing the transistor to a trans-impedance amplifier (TIA, Stanford Research Systems SR570, CA, USA) with a sensitivity ranging between , depending on the relative current magnitude, drift over time and the resolution of the data acquisition system (NI BNC 2110 and NI USB 6259). During transient analysis the signals were high-pass filtered at 1Hz unless specified and low-pass filtered at 3 KHz before sampling to reduce the aliasing effects. Further analysis and filtering was done through custom software. Before every measurement, the transconductance observed from both the CG and SG was recorded in order to calibrate the sensitivity and accurately estimate. For the devices used, observed from the SG was found to range between ~0.01 − 0.3mA/V, limited by parasitics and interface characteristics after repeated cleaning and lysine addition. Impedance measurements monitored the small-signal transistor gain as a function of frequency. The AC sinusoid was supplied by a function generator (Stanford Research Systems DS345, CA, USA) through the Ag/AgCl reference electrode (Warner instruments, USA), while the DC bias (quiescent point) was set by the CG independently (Keithley 2400, USA). The transistor output was fed to a lock-in amplifier (LIA, Stanford Research Systems SR844, CA USA) through the TIA, hence monitoring the root-mean-square voltage as a function of frequency. The sensitivity of the TIA was adjusted so as to avoid saturating the LIA input and was operated under the high bandwidth mode.

The 3dB bandwidth of the CνMOS transistors lie between 10KHz and 300KHz (W/L~1.5) (Fig. S2) with buffer present. The limiting capacitances in our system are the FG to bulk capacitance, the CG capacitance as well as the line capacitances connecting the MOSFET's source and drain to the bond-pads. The line capacitance to the bond pads, along with measured values of S/D capacitance yield a bandwidth of ~10KHz for a 100pF line capacitance and 100KHz for a 50 pF line capacitance. Action potentials will not be attenuated by this filter as the pole is far beyond the bandwidth of interest. Secondly, surface potentials (dc values) are not divided across these impedances as the SG to FG and FG to bulk appear in parallel and coupling through the polymer encapsulation is very weak.

| 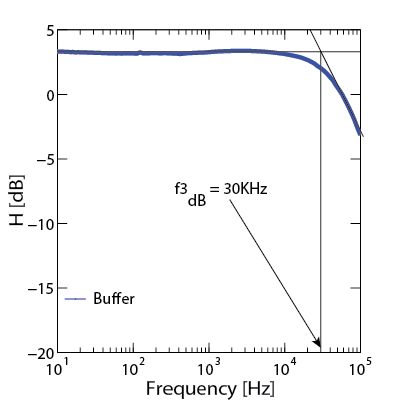 |
| --- |

Figure S2. Bode response of the CνMOS. Notice the first pole occurs at approximately 30kHZ.

**CνMOS Detection Principles**

In CνMOS, the FG voltage is set by the weighted sum of the potentials across all its coupled capacitors (Eq. 1) [Fig. 1(e)]. The input gates have different overlap areas to the FG, which leads to capacitive amplification of the recorded signal. and are the FG-to-source capacitance, FG-to-drain capacitance and total capacitance on FG, respectively. Here represents the static charge stored on the FG. is additionally described as and reflects the change in surface charge through .

(1)

A small change in causes the threshold voltage measured from the CGto be scaled by the amplification factor (). This parameter can be independently tuned with respect to the channel active area which sets the transconductance and frequency response. The nMOS transistor threshold voltage is usually at ~0.8V as measured from the FG, which is normally fixed in foundry. Independent CG control could help alleviate the need to set such large biases on the reference electrode but still maintain the transistor above and ensure high . Furthermore, it can be used to overcome sensor mismatch by pixel level re-biasing or tunneling static charge on and off the FG^1^, reducing the need for complex global calibration circuitry^2^.

Prior to every experiment, the reference electrode was used to calibrate the transconductance observed from the SG interface to extract the capacitive ratio. During the quasi-static and transient measurements the immobilized cells act as independent current sources i.e., they either secrete charge or give rise to ionic currents upon stimulation. Protons and molecules released during this secretory process can bind to the exposed hydroxyl groups on the SG surface and shift. Ionic currents, however, in conjunction with the cell-transistor cleft resistance give rise to a transient voltage in the vicinity of the SG surface. A change in modulates which modulates and hence the output current. It is important to note that with non-faradaic detection the ionic charges of the secreted molecules are not consumed as opposed to the oxidation charge in amperometric detection but can transiently bind to the SG surface or remain within the cell-transistor cleft until they slowly diffuse out. Impedance spectroscopy monitors a shift in the cell’s passive properties via the transfer function (see supporting information). Maintaining constant DC readout current through CG feedback enables pure capacitive detection, as is held constant.

**Impedance Models**

| 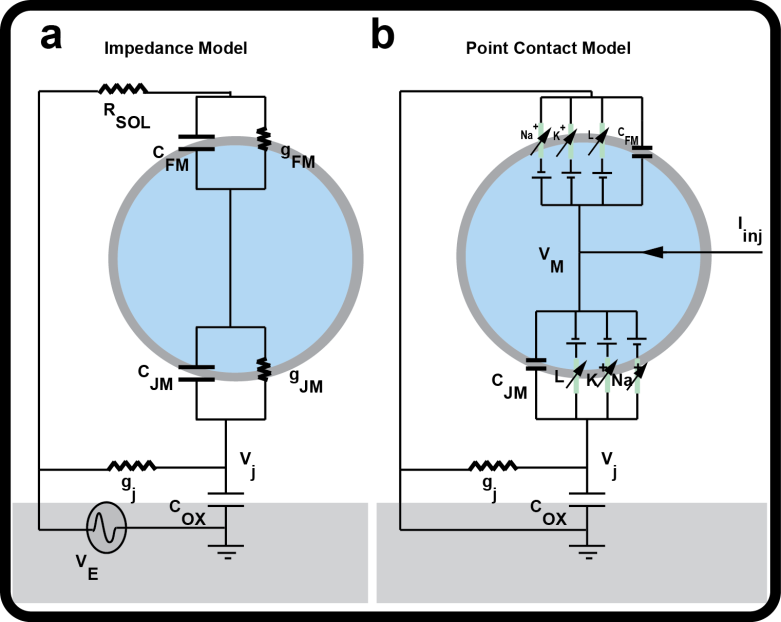 |
| --- |

*Figure S3. (a) Impedance model of the cell-transistor interface and (b) the point contact model describing the methodology to simulate the ionic waves in the cell transistor cleft.*

The equivalent circuit shown in Figure S3(a) is used for impedimetric analysis when working both ISFET and CνMOS. With the ISFET, $C_{\text{OX}}$ serves as the gate capacitance of the transistor while with the CνMOS, using the split excitation technique, the CG serves as AC ground and the overall $C_{\text{OX}}$is given by where $C_{\text{tun}}$is the tunnel oxide capacitance and $C_{\text{tun}}$ is the sensing gate capacitance. The transfer function,$H(j\omega)$ for the circuit shown in Fig. S1(a) can be given by,
Neglecting$g_{\text{FM}}$and$g_{\text{JM}}$ , the first zero and the first two poles can be determined as,

Where $C_{\text{Meff}}$is the parallel combination of $C_{\text{JM}}$and$C_{\text{FM}}$. If $R_{\text{sol}}$is negligible, further simplifications directly reflect the influence of $C_{\text{Meff}}$and$g_{j}$.

***Stability of drain current prior to stimulation and after stimulation***:

In order to demonstrate the stability of the drain current prior to stimulation we show below the first 40 seconds of recording (Fig. 4 main manuscript) of chromaffin cell stimulation (Fig. S4). Notice how the drain current is extremely stable prior to stimulation with high KCl.

| 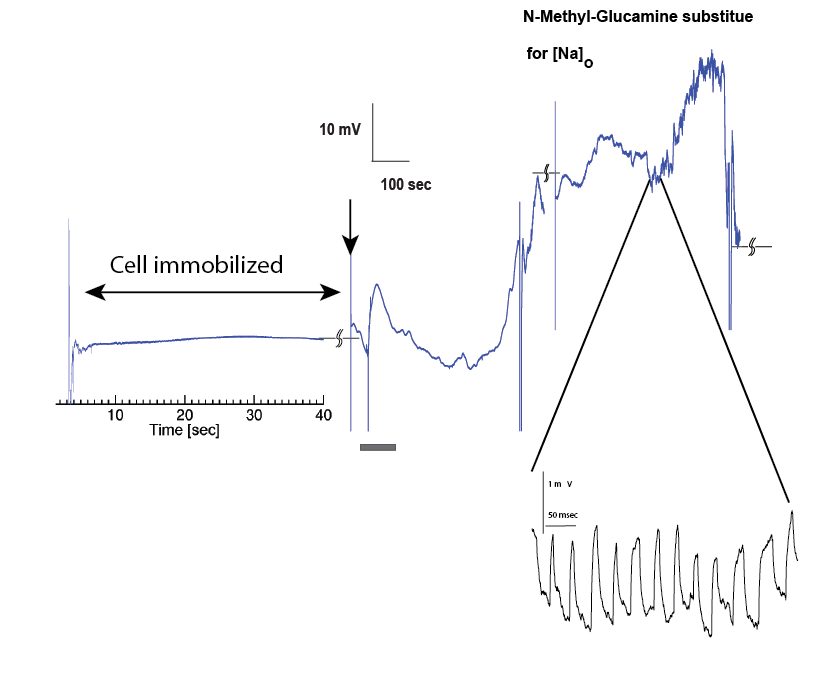 |
| --- |

*Figure S4. stability of the drain current prior to stimulation with cells immobilized on the sensing gate surface. Notice how, upon stimulation (grey bar) there is a sudden change and subsequent rise in drain current indicating positive charge impingement on the sensing gate surface. Clear rhythmic (inset) fluctuations in drain current are observed approximately few minutes into stimulation.*

Shown below (Fig. S5a) is the first 300 seconds of recording (refer Fig 3 in main manuscript) of

| | 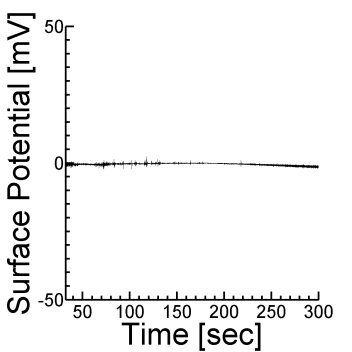  a  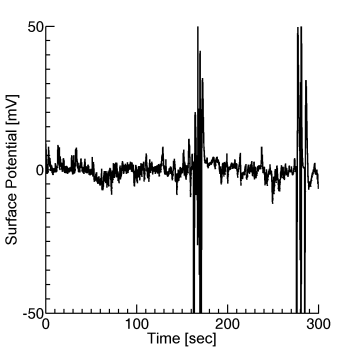  b |  | | --- | --- | | 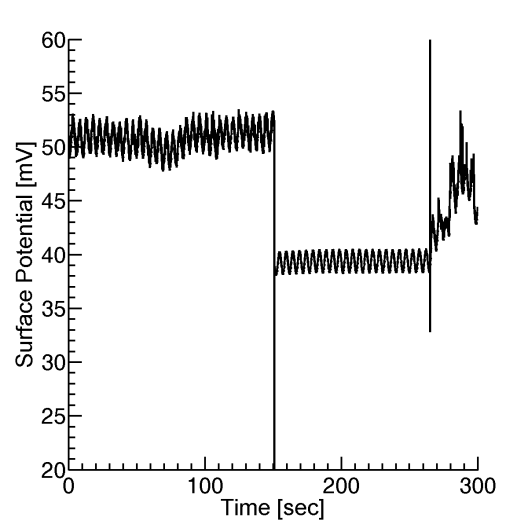  Immobilized cells  Gain change to avoid TIA saturation  Stimulation  c |
| --- | --- | --- | --- |

*Figure S5. (a) surface potential change prior to stimulation (b) surface potential change after stimulation with antigen. The two large artifacts indicate the introduction of buffer media which does not stop activity. (c) Transient recording showing the increase in surface potential upon antigen stimulation*

RBL-2H3 mast cell stimulation prior to addition DNP-BSA (Fig. S5b). The experiment clearly shows that the drain current fluctuations observed are a consequence of mast cell degranulation. Figure S5c further depicts an experimental observation where the drain current is recorded just prior to addition of the stimulant, which clearly shows the stability in the drain current prior to eliciting exocytosis.

References

1 Jayant, K. *et al.* Programmable ion-sensitive transistor interfaces. I. Electrochemical gating. *Physical Review E* **88**, 012801 (2013).

2 Eversmann, B. *et al.* A 128 &times; 128 CMOS biosensor array for extracellular recording of neural activity. *Solid-State Circuits, IEEE Journal of* **38**, 2306-2317 (2003).
